# Using ear molding to correct auricular helix adhesion deformity

**DOI:** 10.3389/fped.2022.990629

**Published:** 2022-11-15

**Authors:** Lili Chen, Chenlong Li, Aijuan He, Ying Chen, Hua Tong, Yaoyao Fu, Tianyu Zhang

**Affiliations:** ^1^ ENT institute, Eye & ENT Hospital, Fudan University, Shanghai, China; ^2^ Department of Facial Plastic and Reconstructive Surgery, Eye & ENT Hospital, Fudan University, Shanghai, China; ^3^ NHC Key Laboratory of Hearing Medicine, Fudan University, Shanghai, China

**Keywords:** ear molding, helix adhesion, ear anomaly, outcome, long-term

## Abstract

**Objectives:**

This study examined the effectiveness of Byrd's EarWell system for the treatment of auricular helix adhesion.

**Methods:**

The newborns with helix adhesion were treated with ear molding. The photos of pinna were taken before, during, and after the treatment. The immediate and long-term outcomes, as well as the complications, were assessed by two independent plastic surgeons.

**Study design:**

A retrospective study. Data on family history, neonatal weight, gestational age, delivery method, laterality, gender, age of initiating treatment, medical comorbidities, duration of treatment, and follow-up time were collected.

**Study site and period:**

From 2019 to 2021, infants treated with the EarWell System in the Eye and ENT Hospital of Fudan University were enrolled in this study.

**Results:**

A total of 46 newborns (66 ears) with helix adhesion were included. The average onset time of treatment was 4.57 ± 3.63 weeks. The average duration of treatment was 7.40 ± 2.05 weeks. 97.0% ears’ (64/66) immediate results were excellent or good. During long-term follow-up, 95.5% ears achieved excellent or good outcomes. Age of initiation treatment significantly affected immediate (*p *= 0.001) and long-term (*p *= 0.004) outcomes.

**Conclusions:**

EarWell System was an effective method to correct auricular helix adhesion. Using this approach, patients with helix adhesion could avoid surgeries. Age of initiation treatment was the predictor of successful correction.

## Introduction

Ear anomalies are common in newborns, with the incidence rate ranging from 15% to 50% (1, 2). An abnormal auricular appearance can cause mental distress. Some studies have found that children with ear anomalies would experience more anxiety, negative self-perception, and interpersonal problems (3–5). So the treatment of ear anomalies is very important for children's psychosocial health (6, 7).

In the past, ear anomalies could only be corrected with surgery. The helix adhesion is a type of mild auricular malformation. Due to the adhesion, the chondrocutaneous component is mildly defective. Scapha skin flap technique and inlay cartilage graft technique are used to correct helical adhesion (8). The potential complications of surgery included wound infection, hemorrhage, and anesthesia-related risks (9). Donor-site deformities, pigmentation of skin grafts, and scarring of the postauricular–mastoid region are still of concern (8). What is more, children could not undergo otoplastic surgeries until they are 5 or 6 years old (10). It has been reported that as age increases, an increasing number of patients experience mood disorders. Thus, it is very important to find a proper treatment that could be initiated at a young age (4).

Recently, the EarWell system, a non-invasive treatment, has been demonstrated to treat neonatal abnormal ears safely and effectively (11, 12). Auricular deformities, as well as some sorts of mild ear malformations, could benefit from this correction system. Nonsurgical methods might be effective for correction auricular adhesion malformation. This study was designed to examine the effectiveness of Byrd's ear molding technique for auricular helix adhesion.

## Patients and methods

### Definition of helix adhesion

**Figure 1:**
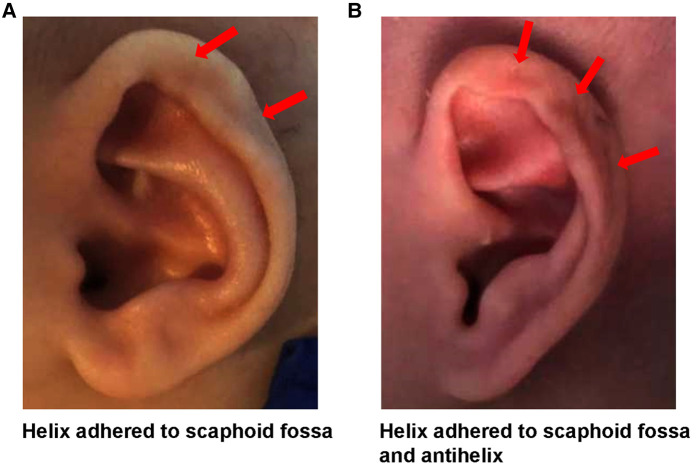
Auricular adhesion anomaly.

Based on the observation of Odili (13) and Chul Park (8), we proposed a modified definition of helix adhesion. Helix adhesion is present when the helix is partially adherent to the scaphoid fossa or antihelix, and there is no intervening layer of skin between the two layers of cartilage (Figure 1). When present, it creates a mild kink in the normal curvature of the helical rim which may consider as less cosmetically pleasant. Due to the adhesion, the cartilage and skin components were slightly defective.

### Patient selection

This retrospective study was approved by the intuitional review board of the Eye and ENT Hospital of Fudan University— a high-volume ear reconstruction center in China. The written informed consent was obtained before starting standard molding treatment. Only infants with a solitary helical adhesion and undergoing external splinting with EarWell system according to Byrd's method (14, 15) met inclusion criteria. Infants who fail to complete molding treatment would be excluded. Between May 2019 and May 2021, a total of 46 infants were enrolled in this study. Demographic and clinical data collected included family history, neonatal weight, gestational age, delivery method, laterality, gender, age at the time of initiating treatment, medical comorbidities, duration of treatment, and date of the last follow-up. Ear photos were taken before, during, and after treatment. A retrospective parents’ satisfaction survey was completed after the last follow-up.

### Treatment process

At the first visit, the molding device was placed by a senior surgeon carefully. Patients were asked to follow up weekly. During treatment, we would adjust the device if necessary. If any complications occurred, the treatment was suspended for 2 to 3 days and local use of antibiotic ointment. Once the skin healed, the ear would continue molding. The complications would be carefully checked and addressed. Outpatient treatment continued for 2 weeks after achieving ear normalization.

Immediate results were assessed by comparing the pre-treatment photos with images that were taken immediately after the infants completed treatment. Ears followed for more than 6 months were considered as “long-term” outcomes. To evaluate the immediate and long-term (≥ 6 months after treatment end) effectiveness, photographs of each ear were taken and compared by two independent and experienced plastic surgeons. Outcomes were graded based on the standards proposed by Byrd (1) (see Supplementary Figure S1). If the two surgeons had disagreements, a third, independent, plastic surgeon was consulted. None of these three surgeons was involved in the treatment process. Ears which were graded excellent or good were considered to achieve successful results.

### Statistical analysis

SPSS software (version 20.0; IBM, New York) was used for statistical analyses. Continuous variables were described as means and standard deviations (SD), and categorical variables were described as frequencies and percentages. Chi-square test was used to calculate *p* values. A value of *p *< 0.05 was considered statistically significant.

## Results

### Patient characteristics

A total of 46 infants with 66 ears were recruited. The basic patient demographics were listed in Table 1. There were 33 (71.7%) newborns who initiated ear molding before 6 weeks of age. Only 4.4% (4/46) patients started EarWell system treatment after 3 months of age. Three (6.5%) cases were premature. Meanwhile, their weights were less than 2500 g. More than half (58.7%) of the babies were born *via* natural birth. The number of the male and the female was very close (24 vs. 22). 18 patients were found with a normal ear, and an abnormal ear with helical adhesion. 8 patients had a helix adhesion ear at one side, and an abnormal ear of other type on the other side. Almost half patients (43.5%, 20/46) had helix adhesion ear on both sides. None of them had a family history of helical adhesion. The average duration of treatment was 7.40 ± 2.05 weeks, and the median treatment time was 7.00 weeks (range: 3.71–14.14 weeks). Follow-up time was defined as the number of days between the time of last visit and the end of treatment, and the average follow-up time was 14.60 ± 6.88 months, and the median follow-up time was 13.27 weeks (range: 6.10–32.8 weeks).

**Table 1:** Clinical characteristics of the 46 newborns with helix adhesion.

Characteristics	No. (%)
Age of initiation treatment	
≤6 wk	33 (71.7)
6 wk–3 m	11 (23.9)
≥3 m	2 (4.4)
Gestational age, wk	
28–37	3 (6.5)
37–42	42 (91.3)
≥42	1 (2.2)
Neonatal weight, g	
<2500	3 (6.5)
2500–4000	40 (87.0)
≥4000	3 (6.5)
Delivery method	
Natural birth	27 (58.7)
Cesarean delivery	19 (41.3)
Gender	
Male	24 (52.2)
Female	22 (47.8)
Affected ears	
Unilateral	18 (39.1)
Bilateral	
Both helix adhesion	20 (43.5)
Helix adhesion + Other type	8 (17.4)
Duration of treatment, wk	
Mean ± SD	7.40 ± 2.05
Median (range)	7.00 (3.71-14.14)
Follow-up time, m	
Mean ± SD	14.60 ± 6.88
Median (range)	13.27 (6.10-32.80)

wk, weeks; m, months; g, gram; SD, standard deviation.

**Figure 2:**
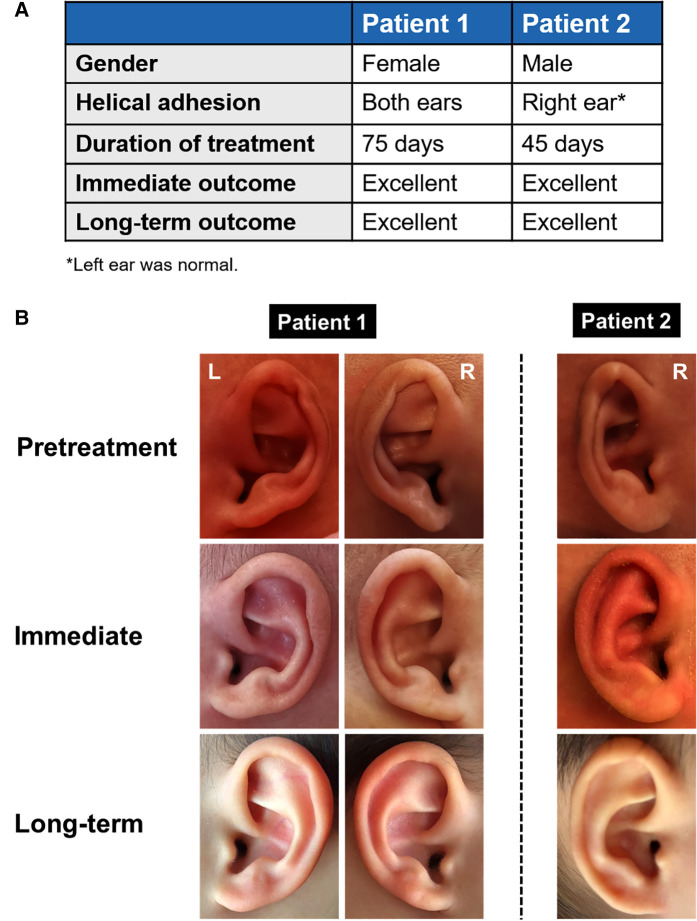
One dizygotic twin pair with helix adhesion.

It was interesting that we found one dizygotic twin pair (Figure 2). They both suffered from helical adhesion. They were premature with birth weights less than 2500 g and started ear molding at 34 days of birth. This implied that genetic factors might contribute to helical adhesion.

### Immediate treatment outcomes

Ears with good or excellent outcomes were considered successful in terms of corrections. Most ears (58/66, 87.9%) were graded excellent. Six ears were good, and one ear was fair. No ears' immediate results were poor. Therefore, the immediate success rate was 97.0 percent (64/66, Table 2). The 2 ears with fair immediate outcomes were from a female patient, with 2500 g birth weight. This patient initiated correction at 112 days (exceeding 3 months after birth) and was treated for 50 days (7.14 weeks). This patient was found to have ulcer and dermatitis simultaneously.

**Table 2:** Immediate and long-term outcomes of the 66 ears with helix adhesion.

Characteristics	Immediate outcomes^a^				Long-term outcomes				
	Excellent	Good	Fair	Success rate^b^	Excellent	Good	Fair	Poor	Success rate^b^
Age of initiation treatment									
≤ 6 wk	45	5	0	100%	44	6	0	0	100%
6 wk–3 m	12	1	0	100%	8	4	1	0	92.3%
≥ 3 m	1	0	2	33.3%	1	0	0	2	33.3%
Gestational age, wk									
28–37	3	1	0	100%	4	0	0	0	100%
37–42	53	5	2	96.7%	47	10	1	2	95.0%
≥ 42	2	0	0	100%	2	0	0	0	100%
Neonatal weight, g									
<2500	3	1	0	100%	4	0	0	0	100%
2500–4000	50	5	2	96.5%	46	9	0	2	96.5%
≥ 4000	5	0	0	100%	3	1	1	0	80.0%
Side									
Left	30	5	1	97.2%	27	7	1	1	94.4%
Right	28	1	1	96.7%	26	3	0	1	96.7%
Affected ears									
Unilateral	17	1	0	100%	15	3	0	0	100%
Bilateral									
Both helix adhesion	34	4	2	95.0%	31	6	1	2	92.5%
Helix adhesion + Other type	7	1	0	100%	7	1	0	0	100%
Gender	29	5	0	100%	25	8	1	0	97.1%
Male	29	1	2	93.8%	28	2	0	2	93.8%
Female									
Complications	7	1	2	80.0%	5	3	0	2	80.0%
Yes	51	5	0	100%	48	7	1	0	98.2%
No									
Delivery method	34	4	0	100%	30	7	1	0	97.4%
Natural birth	24	2	2	92.9%	23	3	0	2	92.9%
Cesarean delivery	58	6	2	97.0%	53	10	1	2	95.5%

wk, weeks; m, months; g, gram.

^a^ No ears’ immediate results were poor.

^b^ The ears which were graded excellent or good were considered to achieve successful results.

**Figure 3:**
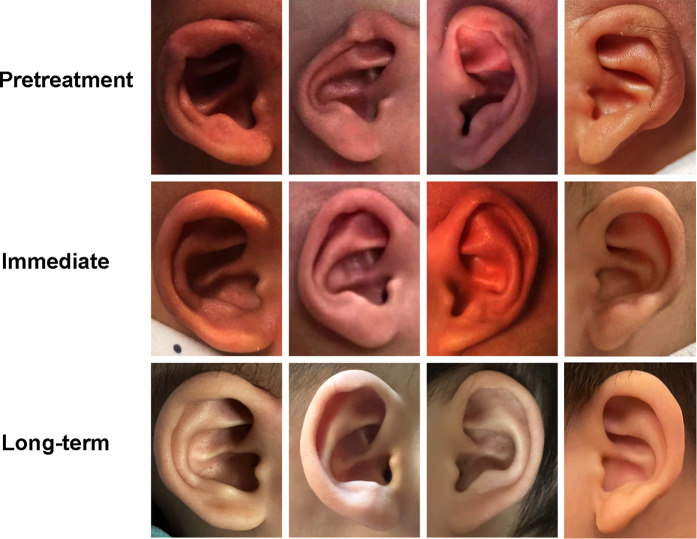
Examples of successful correction for helical adhesion.

Examples of helix adhesion ears treated using the EarWell System were shown in Figure 3. Ten (15.2%, 10/66) ears were found with dermatitis and pressure ulcers.

### Long-term treatment outcomes

When evaluating the long-term effectiveness of ear molding technique for helix adhesion, 53 ears (80.3%) were graded excellent, and 10 (15.2%) were graded good (Table 2). So the long-term success rate was 95.5 percent (63/66). One ear's long-term grading was fair. This ear was from a male patient. He had helix adhesion ears on both sides. This patient initiated correction at 52 days (7.43 weeks) and was treated for 56 days (8 weeks). The immediate outcomes of his 2 ears were excellent. However, these 2 ears fail to maintain normal shape over time. The grading changed from excellent to good (left ear) and from excellent to fair (right ear). We noticed that the 2 ears with fair immediate outcomes got worse during long-term follow-up, and they were graded poor at the last visit. These 2 ears were also from a single person, who initiated molding at the age of 3.7 months after birth.

### Age of initiation treatment affects molding effectiveness

As shown in Table 3, there was a significant difference in the age of initiation treatment between patients with successful and unsuccessful immediate results (*p *= 0.001). Long-term outcomes were also significantly correlated with the age of initiation treatment (*p *= 0.004).

**Table 3:** Age of initiation treatment affecting molding effectiveness for helix adhesion ear.

Variables	Immediate outcome		*P* value	Long-term outcome		*P* value
	Success	Failure		Success	Failure	
Age of initiation treatment			**0** **.** **001**			**0**.**004**
< 3 months	63	0		62	1	
≥ 3 months	1	2		1	2	

### Complications

**Figure 4:**
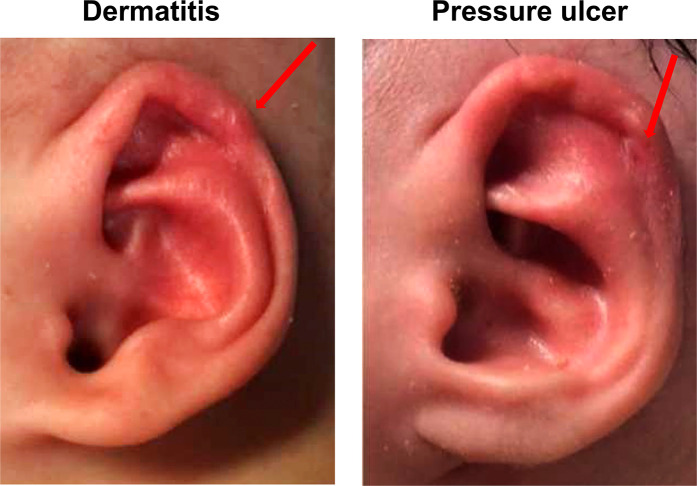
Complications of the EarWell system.

During treatment, the babies were examined weekly. Ten (15.2%) ears experienced complications (Figure 4). Dermatitis and ulcers were observed in 7 and 5 ears (2 ears with the two complications simultaneously), respectively.

### Parents' satisfaction

**Figure 5:**
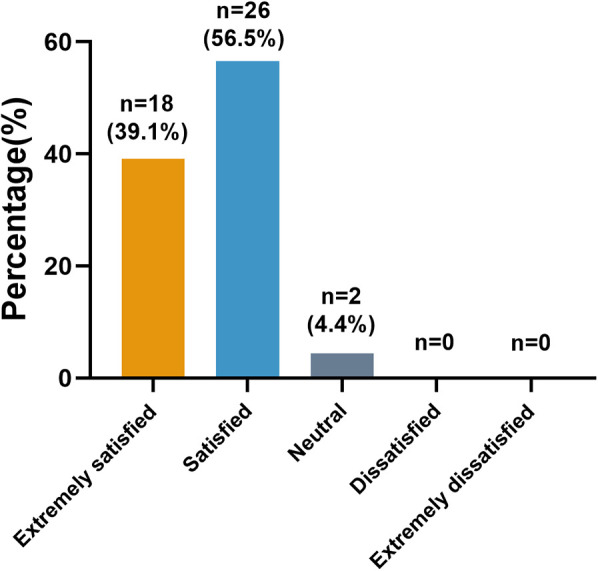
Parents’ satisfaction rates.

Among the 46 infants' parents, 39.1% (18/46) felt extremely satisfied, 56.5% (26/46) satisfied, only 4.4% (2/46) neutral. No dissatisfaction or extreme dissatisfaction with the treatment results was observed (Figure 5).

## Discussion

There have been few previous studies of helical adhesion. In this study, we investigated the use of EarWell Infant Ear Correction System for patients with helical adhesion. We found the immediate and long-term success rates were 97.0% and 95.5%, respectively. The results of this study suggest that EarWell System can have favorable outcomes for helical adhesion. In addition, parent satisfaction with the treatment was also high (95.6%). Our results demonstrated that the age of initiation treatment was a significant predictor of both immediate (*p *= 0.001) and long-term corrections (*p *= 0.004). Infants initiating molding at early age had more stable outcomes. It is strongly advised to commence ear molding as soon as possible to attain an ideal ear shape for patients with helix adhesion.

Ear cartilage can be remolded at an early age, usually no more than 3 months. So it is very important to recognize helical adhesion soon after birth. However, helical adhesion malformations are still poorly identified. From 1986 to 2015, some Japanese literature depicted a group of helical adhesion deformity of upper third auricular cartilage (16–19). Odili and Gault also observed this unusual deformity with helical adhering to the scapha and obliterated helical sulcus. They called this deformity fold-over helical rim (8). Different from lidding ear, there was no skin between the two layers of cartilage because the cartilage adhered tightly or fused. Chul Park (14) noticed similar auricular adhesion malformations and divided them into 3 groups: solitary helical adhesion (the helical cartilage is attached to the anterior scapha cartilage), solitary antihelical adhesion (the posterior scapha cartilage is attached to the posterior conchal wall cartilage), helical and antihelical adhesion. In this study, we defined helical adhesion as a mild malformation which presented as helix adhering to adjacent ear structures, such as scaphoid fossa or antihelix, with or without helix rim pleating. Our definition was more comprehensive and easier to understand and remember, thus it would help physicians identify the helical adhesion malformation more accurately and timely.

Almost all studies of helical adhesion focused on how to correct malformation with surgery. Korean researchers recommend scapha skin flap technique and inlay cartilage graft technique for helical adhesion (14, 20). A technique of composite concha tissue graft

on the transection site of the scapha (21) and a technique of redraping of the helical skin after helical cartilage correction using Tanzer's banner flap technique were also suggested (17, 22). In some cases requiring a conchal cartilage graft, patients were advised to delay the operation until reached 12 years old. In fact, as an invasive treatment, surgery would bring potential complications such as infection, hemorrhage, hypertrophic scar, skin necrosis, and so on. Besides, children usually began to forge a personal identity and a self-concept around the age of 6 years. Treatment delays would make it difficult for children to have normal social interactions, thus affecting their studies and mental health.

Encouragingly, ear molding as a non-invasive technique could correct auricular deformities and slight malformations safely and effectively. The molding could be initiated long before the onset of teasing, bullying, and loss of self-esteem (23). We found some ear molding studies may include several cases of helix adhesion, although these cases were not named helix adhesion, the morphological features of the ear figures indicated that patients were with the helix adhesion deformity (1, 11). However, as these helix adhesion cases were mixed with other deformities, the molding effect of helix adhesion cannot be determined. To the best of our knowledge, this is the first study to focus on the effectiveness of ear mold treatment for patients with helix adhesion malformations, and we found this type of malformation could be well corrected by molding technique. This study aimed to help people recognize the morphological features of helix adhesion and clarify this type of malformation could be early treated by a non-invasive ear correction system. We hope that more clinicians know helix adhesion and that more patients with helix adhesion could receive molding treatment in time and avoid surgical trauma.

There were several limitations of this research. First, it was a retrospective study and the design may have an inherent bias, so the results need to be confirmed by a prospective study. Besides, the conclusions were based on data from a single-center, and need to be validated in multi-center studies. During clinical practice, we found the ear cartilage and skin of neonatal could be extended by mechanical force. We would explore the mechanisms in future study.

## Conclusions

The immediate and long-term outcomes of helix adhesion after molding treatment demonstrated that EarWell System was an effective and safe method. Doctors should recognize helical adhesion accurately and timely, and then help patients initiate molding in the early time to achieve favorable ear shape.

## Data Availability

The raw data supporting the conclusions of this article will be made available by the authors, without undue reservation.
