# Supplementary figures and images for: Using ear molding to correct auricular helix adhesion deformity
Source: Front Pediatr. 2022 Nov 15;10:990629. doi: 10.3389/fped.2022.990629 (PMC9706092; doi:10.3389/fped.2022.990629)

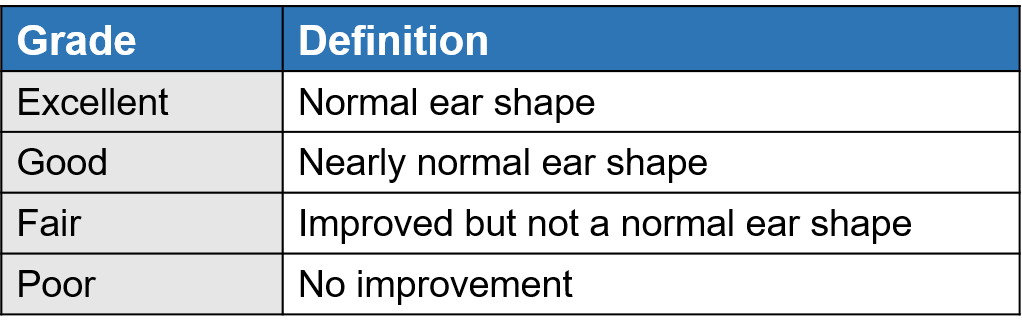

Supplement: Supplementary file 1 [file Image1.tif]
